# HIF1A inhibitor PX-478 reduces pathological stretch-induced calcification and collagen turnover in aortic valve

**DOI:** 10.3389/fcvm.2022.1002067

**Published:** 2022-11-07

**Authors:** Md Tausif Salim, Nicolas Villa-Roel, Booth Vogel, Hanjoong Jo, Ajit P. Yoganathan

**Affiliations:** ^1^School of Chemical and Biomolecular Engineering, Georgia Institute of Technology, Atlanta, GA, United States; ^2^The Wallace H. Coulter Department of Biomedical Engineering, Georgia Institute of Technology and Emory University, Atlanta, GA, United States; ^3^School of Chemistry and Biochemistry, Georgia Institute of Technology, Atlanta, GA, United States

**Keywords:** cyclic stretch, HIF1A, PX-478, aortic valve, calcification

## Abstract

HIF1A is significantly upregulated in calcified human aortic valves (AVs). Furthermore, HIF1A inhibitor PX-478 was shown to inhibit AV calcification under static and disturbed flow conditions. Since elevated stretch is one of the major mechanical stimuli for AV calcification, we investigated the effect of PX-478 on AV calcification and collagen turnover under a pathophysiological cyclic stretch (15%) condition. Porcine aortic valve (PAV) leaflets were cyclically (1 Hz) stretched at 15% for 24 days in osteogenic medium with or without PX-478. In addition, PAV leaflets were cyclically stretched at a physiological (10%) and 15% for 3 days in regular medium to assess its effect of on HIF1A mRNA expression. It was found that 100 μM (high concentration) PX-478 could significantly inhibit PAV calcification under 15% stretch, whereas 50 μM (moderate concentration) PX-478 showed a modest inhibitory effect on PAV calcification. Nonetheless, 50 μM PX-478 significantly reduced PAV collagen turnover under 15% stretch. Surprisingly, it was observed that cyclic stretch (15% vs. 10%) did not have any significant effect on HIF1A mRNA expression in PAV leaflets. These results suggest that HIF1A inhibitor PX-478 may impart its anti-calcific and anti-matrix remodeling effect in a stretch-independent manner.

## Introduction

Heart valve diseases are prevalent in more than 13% of the elderly population aged > 75 years old (1). Aortic stenosis (AS) is one of the most prevalent heart valve diseases, affecting more than 3.4% of the elderly population (2). When untreated, the mortality rate is about 25% per year after the onset of severe symptoms (angina, syncope, heart failure, etc.) (3). AS is characterized by pathological narrowing of the aortic valve (AV) opening during systole, resulting in increased pressure gradient across the valve. The narrowing of AV opening is caused by significantly thicker and stiffer AV leaflets (4). The higher thickness and stiffness in AV leaflets arise from pathological fibrosis and calcification of AV tissue that take place over a period of several years (4). Unfortunately, despite its high prevalence, there is no clear understanding of the underlying mechanism of AS pathogenesis and disease progression (5). As a consequence, there are currently no therapeutic drugs available to treat AS and the only treatment options are either surgical or transcatheter AV replacement (6, 7).

Previously, it was believed that AS is a result of an age-related tissue degeneration process. However, extensive mechanobiological research in the last two decades has shown that mechanical forces play a significant role in the initiation and progression of AV fibrosis and calcification, implying that AS is not a mere manifestation of age-related passive degeneration. Rather, this disease results from an active pathological process spanning several years (8). The AV experiences different types of mechanical force during each cardiac cycle, including tensile stress, bending stress, and shear stress (9). For the healthy functioning of AV leaflets, each of these mechanical forces should remain within their respective optimal (or physiological) ranges. Any chronic (or pathological) deviation in these mechanical forces promotes AV pathogenesis, resulting in AV fibrosis and calcification. Elevated mechanical stretch and low oscillatory shear stress (i.e., disturbed flow) have been shown to promote AV pathogenesis, leading to AV fibrosis and calcification *ex vivo* (10–13). In addition, high mechanical stretch was found to induce significantly higher AV calcification compared to low oscillatory shear stress (14), indicating that elevated stretch has a more prominent effect on AV calcification compared to disturbed flow. Furthermore, elevated circumferential stretch (e.g., 15%) was shown to represent hypertensive condition for AV, whereas physiological level of stretch (e.g., 10%) corresponded to normotensive condition (15). Since hypertension is one of the major risk factors for calcific aortic valve disease (CAVD) (8), this suggests a potential causal link between elevated mechanical stretch and AV calcification.

Hypoxia Inducible Factor 1 (HIF1) is a basic-helix-loop-helix-PAS transcription factor that plays a critical role in activating homeostatic responses to hypoxia (16). HIF1 is a heterodimer consisting of two subunits, HIF1A and HIF1B. It was previously shown that HIF1A protein expression was significantly higher in the calcified areas of stenotic human AVs compared to non-calcified areas (17). In addition, low oscillatory shear stress (i.e., disturbed flow) was found to upregulate HIF1A mRNA and protein expression in human aortic valve endothelial cells (HAVECs) compared to laminar shear stress (i.e., stable flow) (18).

4-[bis(2-chloroethyl)oxidoamino]-L-phenylalanine dihydrochloride, or PX-478, is a specific inhibitor of HIF1A (19). This orally available pharmacological agent was tested in a clinical trial as a potential treatment for metastatic cancer (20). Recently, we showed that PX-478 treatment significantly inhibits AV calcification under static and disturbed flow conditions (18). Furthermore, PX-478 was found to significantly reduce aortic plaque burden in atherosclerotic mice (21).

Since elevated stretch is a more prominent inducer of AV calcification compared to disturbed flow (14), the overall objective of this study was to evaluate the effect of the HIF1A inhibitor PX-478 on AV calcification and collagen turnover, an important pathophysiological marker of AV disease, under high cyclic stretch. In addition, the effect of cyclic stretch on HIF1A mRNA expression was assessed in AV tissue samples. Results from this study could provide significant insight into the functional role of HIF1A and its inhibitor PX-478 in CAVD.

## Materials and methods

### *Ex vivo* cyclic stretch experiments

**FIGURE 1:**
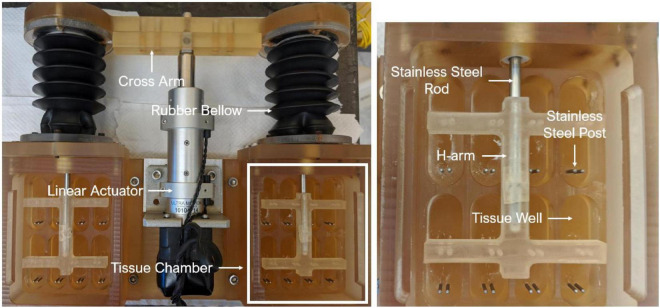
*Ex vivo* cyclic stretch bioreactor.

An *ex vivo* experimental approach was adopted in this study. Briefly, fresh porcine aortic valve (PAV) leaflets were collected from a local abattoir (HOLIFIELD FARMS) and transported back to the laboratory. A rectangular section of 16 mm (circumferential direction) by 8 mm (radial direction) was excised from the belly region of each leaflet, followed by insertion of stainless-steel springs at both circumferential ends. The sprung PAV tissue sections were then mounted in an *ex vivo* cyclic stretch bioreactor (Figure 1), which was previously validated in our laboratory (22).

To assess the effect of cyclic stretch on specific mRNA expression, sprung PAV tissue sections were cyclically (1 Hz) stretched at 10 and 15% in the circumferential direction for 3 days in regular medium. The circumferential direction was chosen for cyclic stretching since PAV tissue is known to be stiffer in the circumferential direction due to preferential alignment of the collagen fibers (23). 10 and 15% were previously shown to be the physiological and pathological levels of circumferential stretch, being representative of normotensive and hypertensive condition, respectively (15). The regular medium comprised of 13.36 g/L DMEM (FISHER SCIENTIFIC), 10% bovine calf serum (FISHER SCIENTIFIC), 3.7 g/L sodium bicarbonate (SIGMA ALDRICH), 50 mg/L ascorbic acid (SIGMA ALDRICH), 2.5% HEPES buffer (FISHER SCIENTIFIC), 1% non-essential amino acid (SIGMA ALDRICH), and 1% antibiotics (FISHER SCIENTIFIC) (10). To simulate stretch-induced calcification *ex vivo*, sprung PAV tissue sections were cyclically (1 Hz) stretched at 15% in the circumferential direction for 24 days in osteogenic medium. The osteogenic medium was used to accelerate the calcification process. It consisted of the regular medium supplemented with 3.8 mM monosodium phosphate (SIGMA ALDRICH), 1 mM β-glycerophosphate (SIGMA ALDRICH), and 10 μM dexamethasone (SIGMA ALDRICH) (11).

### HIF1A inhibitor PX-478

HIF1A inhibitor PX-478 was obtained from MEDKOO BIOSCIENCES. PX-478 was freshly prepared in Dulbecco’s phosphate buffered saline (DPBS) for each supplementation (every 2–3 days) during the course of an experiment. Two different concentrations of PX-478 were used in this study, 50 μM (moderate concentration) and 100 μM (high concentration). The maximum dosage of PX-478 (100 μM) was chosen based on the work done by Villa-Roel et al. (21) The v/v percentage, when PX-478 or PBS were added to the culture medium, was, 0.2 and 0.4% for 50 and 100 μM group, respectively. The concentration of the PX-478 stock solution was 25 mM, as described in Villa-Roel et al. (21).

### Assessment of porcine aortic valve calcification

Porcine aortic valve (PAV) calcification was quantitatively assessed by Arsenazo assay. Briefly, PAV tissue samples were homogenized and digested in 1 M acetic acid (SIGMA ALDRICH) solution for 24 h. The resulting supernatant was then reacted with Arsenazo III dye (POINTE SCIENTIFIC) to determine the amount of calcium in each sample. In addition, Alizarin Red staining was used to qualitatively assess PAV calcification. Briefly, 10-μm frozen PAV tissue sections were hydrated and incubated in 2% Alizarin Red (SIGMA ALDRICH) solution (pH = 4.1–4.3) for 30 s to 1 min. Alizarin Red binds to calcium and positive staining is identified as orange or red in color. Furthermore, ImageJ software was used to quantify the amount of positively stained area as a percentage of total tissue area.

### Assessment of porcine aortic valve collagen turnover

The degree of collagen turnover was assessed by quantifying the ratio of immature to mature collagen in PAV tissue samples. Picrosirius Red staining was performed to identify mature (red) and immature (green and yellow) collagen fibers (24). Briefly, 10-μm frozen PAV tissue sections were hydrated and incubated in Picrosirius Red (saturated picric acid) solution (SIGMA ALDRICH) for 1 h, followed by a wash in 0.5% acidified water. The stained PAV tissue samples were then imaged using a polarized light microscope. ImageJ software was used to quantify the amount of mature and immature collagen in PAV leaflets.

### RNA isolation and quantitative polymerase chain reaction

An RNA isolation kit (ZYMO RESEARCH) was used to isolate total RNA. cDNA was synthesized using a reverse transcription kit (QIAGEN). qPCR was carried out in triplicates using a 96-well Real-Time PCR system (THERMO FISHER SCIENTIFIC) and 18S was used as the housekeeping gene. The relative mRNA expression was calculated using the ΔCT method (25).

### Statistical analysis

Data are presented as mean ± standard error of mean (SEM). Independent samples *t*-test was used to compare two independent and normally distributed data sets, whereas non-normally distributed data sets were statistically compared using non-parametric Mann-Whitney *U* test. IBM SPSS Statistics software was used to conduct all statistical comparisons. A *p*-value of 0.05 or less was considered to indicate statistical significance. In addition, effect size (Hedges’ *g*-value) was calculated for each statistical comparison, where Hedges’ *g*-values of 0.2, 0.5, and 0.8 were considered to indicate small, medium and large effect sizes, respectively (26).

## Results

### Effect of high concentration PX-478 on porcine aortic valve calcification under pathological (15%) stretch

**FIGURE 2:**
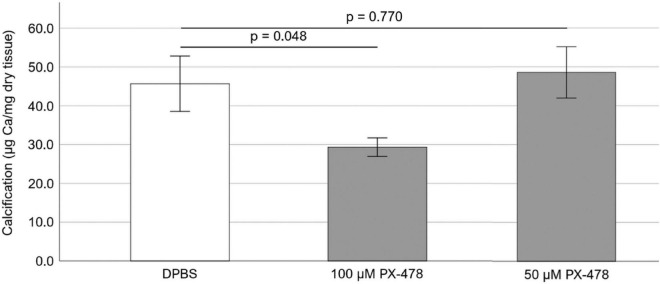
Effect of 50 and 100 μM PX-478 on porcine aortic valve (PAV) leaflet calcification after 24 days of 15% stretch in osteogenic medium, as determined by Arsenazo assay (*n* = 8).

Freshly obtained PAV leaflets were cyclically (1 Hz) stretched at pathological (15%) level for 24 days in osteogenic medium. The osteogenic medium was replaced every 2–3 days and supplemented with either DPBS or 100 μM PX-478. It was found that treatment with 100 μM PX-478 resulted in a significant decrease (*p* = 0.048) in PAV leaflet calcification (35.8% decrease with Hedges’ *g*-value of 1.08) under 15% stretch in osteogenic medium compared to the control case, as determined by Arsenazo assay (Figure 2).

### Effect of moderate concentration PX-478 on porcine aortic valve calcification under pathological (15%) stretch

To test whether a lower concentration of PX-478 could still inhibit PAV calcification, freshly obtained PAV leaflets were cyclically (1 Hz) stretched at pathological (15%) level for 24 days in osteogenic medium, supplemented with either DPBS (control) or moderate concentration (50 μM) PX-478 every 2–3 days. Treatment with 50 μM PX-478 did not have any significant effect (*p* = 0.770) on PAV leaflet calcification (6.4% increase with Hedges’ *g*-value of 0.15) under 15% stretch in osteogenic medium compared to the control case, as determined by Arsenazo assay (Figure 2).

**FIGURE 3:**
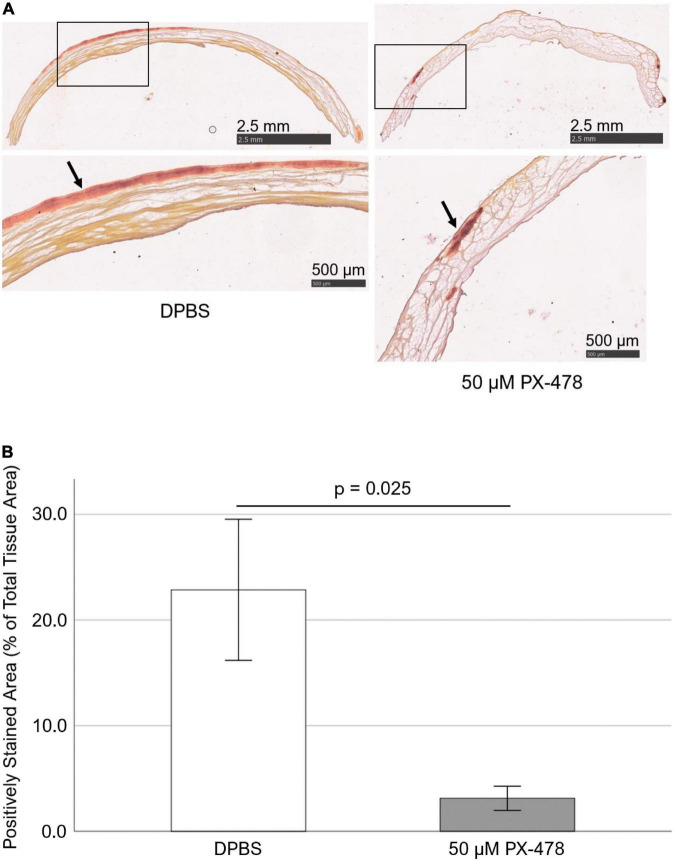
PX-478 inhibits porcine aortic valve (PAV) calcification in response to cyclic stretching. **(A)** Representative Alizarin Red staining images of PAV leaflets (top row: lower magnification, bottom row: higher magnification) after 24 days of 15% stretch in osteogenic medium. Arrows indicate areas of calcification. **(B)** Quantification of Alizarin Red staining images of PAV leaflets after 24 days of 15% stretch in osteogenic medium (*n* = 5–7).

In addition, Alizarin Red staining was used to qualitatively assess the effect of 50 μM PX-478 on PAV leaflet calcification under 15% stretch in osteogenic medium. Interestingly, treatment with 50 μM PX-478 significantly lowered positive staining (i.e., calcification) compared to the control case [Figure 3A]. This was confirmed by the quantification of positively stained area as a percentage of total tissue area (using ImageJ software), which showed a significant reduction (*p* = 0.025) with 50 μM PX-478 treatment (86.3% decrease with Hedges’ *g*-value of 1.43) compared to the control case [Figure 3B]. These results show that 50 μM PX-478 inhibits PAV leaflet calcification.

### Effect of moderate concentration PX-478 on porcine aortic valve collagen turnover under pathological (15%) stretch

**FIGURE 4:**
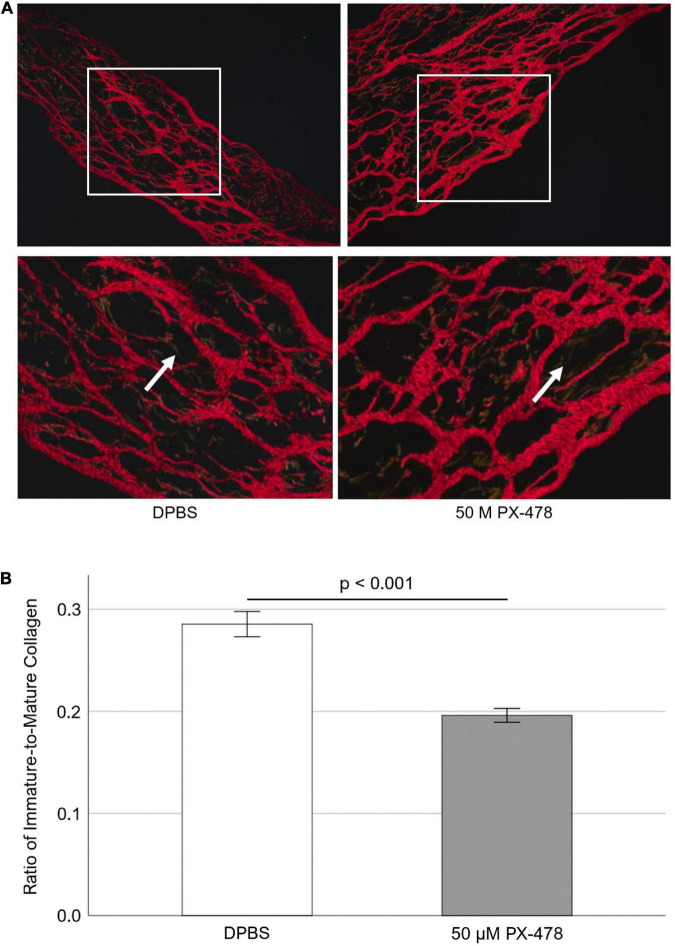
**(A)** Representative Picrosirius Red staining images of porcine aortic valve (PAV) leaflets after 24 days of 15% stretch in osteogenic medium. Red fibers indicate mature collagen; green and yellow fibers indicate immature collagen (white arrows). **(B)** Quantification of Picrosirius Red staining images of PAV leaflets after 24 days of 15% stretch in osteogenic medium (*n* = 7). Each sample comprises of multiple images representing multiple locations (minimum 5 images per sample).

To investigate whether moderate concentration PX-478 could inhibit PAV collagen turnover, freshly obtained PAV leaflets were cyclically (1 Hz) stretched at pathological (15%) level for 24 days in osteogenic medium, supplemented with either DPBS (control) or moderate concentration (50 μM) PX-478 every 2–3 days. Picrosirius Red staining was performed to identify mature (red) and immature (green and yellow) collagen fibers under polarized light [Figure 4A]. ImageJ software was used to quantify PAV collagen turnover as the ratio of immature to mature collagen. It was found that treatment with 50 μM PX-478 significantly decreased (*p* < 0.001) the ratio of immature to mature collagen fibers in PAV leaflets (31.3% decrease with Hedges’ *g*-value of 0.97) under 15% stretch in osteogenic medium compared to the control case [Figure 4B]. This result suggests that 50 μM PX-478 inhibits pathological collagen turnover in PAV leaflets under 15% stretch in osteogenic medium.

### Effect of cyclic stretch on HIF1A mRNA expression in porcine aortic valves

**FIGURE 5:**
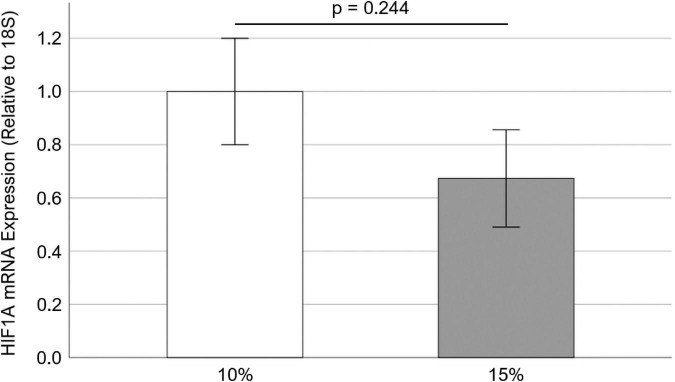
Effect of cyclic stretch on HIF1A mRNA expression in porcine aortic valve (PAV) leaflets (*n* = 10).

Since HIF1A inhibitor PX-478 could inhibit PAV leaflet calcification and collagen turnover, it was tested to see how cyclic stretch modulates HIF1A mRNA expression in PAV leaflets. Freshly obtained PAV leaflets were cyclically (1 Hz) stretched at physiological (10%) and pathological (15%) levels for 3 days in the regular medium. We found that there was no significant difference (*p* = 0.244) in HIF1A mRNA expression (32.7% decrease with Hedges’ *g*-value of 0.54) between 10 and 15% stretch (Figure 5), which may be due to a short half-life of PX-478 in the face of its constitutively high transcription.

## Discussion

In this study, we investigated the effect of HIF1A inhibitor PX-478 on PAV calcification and collagen turnover under pathological 15% stretch. We found that high concentration (100 μM) PX-478 could significantly inhibit PAV calcification under 15% stretch (Figure 2). However, this anti-calcific effect of PX-478 was diminished at moderate 50 μM concentration (Figure 2), although it could still impart some level of inhibitory effect on PAV calcification (Figure 3). The discrepancy between Arsenazo assay and Alizarin Red staining results may be due to the different calcification detection mechanism of these two methods. Arsenazo assay shows the degree of calcification in the whole sample quantitatively whereas Alizarin Red staining helps in detecting localized patterns of calcification qualitatively. Calcified areas of stenotic human AVs were found to express significantly higher level of HIF1A protein compared to non-calcified areas (17). HIF1A inhibitor PX-478 was previously shown to inhibit PAV calcification under static and low, oscillatory shear conditions (18). In addition, PX-478 was found to significantly reduce heterotopic ossification of soft tissue in a burn/tenotomy mouse model (27). Bone Gamma-Carboxyglutamate Protein (BGP) overexpression was shown to induce vascular calcification in a HIF1A-dependent manner (28). Similarly, it was found that HIF1A expression was significantly higher in calcifying aortas of nephrectomized male Wistar rats on high-mineral diet along with calcitriol supplementation compared to nephrectomized ones on normal diet (29). Advanced glycation end products (AGEs) were shown to enhance vascular smooth muscle cell (VSMC) calcification by activating the HIF1A-Pyruvate Dehydrogenase Kinase 4 (PDK4) pathway and inhibition of PDK4 expression significantly reduced VSMC calcification (30). Furthermore, it was found that hypoxia (5% O_2_) induced osteogenic differentiation of VSMCs in a HIF1A-dependent and mitochondria-derived reactive oxygen species (ROS)-dependent manner compared to normoxic (21% O_2_) condition (31). Interestingly, Interferon-γ (IFN-γ) and lipopolysaccharide (LPS), in combination, were shown to induce calcification of human aortic valve interstitial cells (HAVICs) under normoxic condition by activating the Signal Transducer and Activator of Transcription 1 (STAT1)/HIF1A pathway (32). Hypoxic (13% O_2_) culture of aged (> 2 years) PAV tissue resulted in significant upregulation of the expression of Matrix Metalloproteinase 9 (MMP9)-Neutrophil Gelatinase-Associated Lipocalin (NGAL) complex and fragmentation of elastic fibers (in the ventricularis layer) compared to fresh ones (33). In addition, hypoxia (13% O_2_) induced the formation of ectopic, nascent elastic fibers in the fibrosa layer of aged (> 2 years) PAV tissue compared to fresh and normoxic (20% O_2_) ones, implying pathological alteration in elastic matrix composition (33).

Moderate concentration (50 μM) PX-478 was found to significantly inhibit PAV collagen turnover under 15% stretch in osteogenic medium (Figure 4). The collagen fiber architecture was previously shown to be significantly altered in calcified human AVs compared to healthy ones (34). Furthermore, immature collagen was found to be associated with regions of microcalcification in atherosclerotic plaques (35). In a recent study (21), it was shown that PX-478 significantly reduced aortic plaque burden in two chronic mouse models of atherosclerosis.

Interestingly, it was observed that there was no significant difference in HIF1A mRNA expression in PAV leaflets between 10 and 15% stretch (Figure 5). Low, oscillatory shear stress (i.e., disturbed flow) was previously shown to upregulate HIF1A mRNA and protein expression in HAVECs compared to laminar shear stress (i.e., stable flow) (18). However, it was found that O_2_ diffusion coefficient was essentially similar in PAV leaflets between 4.5 and 10.5 kPa (i.e., different degree of leaflet stretching) under normoxic (20% O_2_) condition (36). Furthermore, it was shown that there was no significant difference in local O_2_ diffusion within the PAV tissue under 4.5 and 10.5 kPa pressure (36). As HIF1A expression is directly related to O_2_ diffusion (16), it can be argued that no significant difference in HIF1A mRNA expression between 10 and 15% was possibly due to similar O_2_ diffusion under these stretch conditions. Specifically, elevated cyclic stretch (15% vs. 10%) would result in higher tissue thinning, leading to a lower O_2_ diffusion path and lower HIF1A expression in PAV tissue. This was possibly observed as non-significant 32.7% decrease in HIF1A mRNA expression at 15% stretch compared to 10% in PAV leaflets (Figure 5).

Hence, these results indicate that high concentration (100 μM) PX-478 significantly inhibits PAV calcification under 15% stretch in osteogenic medium, while moderate concentration (50 μM) PX-478 imparts some inhibitory effect on PAV calcification. Nevertheless, 50 μM PX-478 can significantly reduce PAV collagen turnover under 15% stretch in osteogenic medium. Since cyclic stretch (10 and 15%) does not significantly affect HIF1A mRNA expression in PAV leaflets, this implies that HIF1A inhibitor PX-478 may inhibit PAV calcification and collagen turnover in a stretch-independent manner (at least within the range of 10–15% stretch). Unfortunately, as HIF1A protein is highly unstable under normal oxygen condition, it could not be measured by western blot. In addition, HIF1A mRNA was not measurable after 24 days of cyclic stretching. Further studies need to be conducted to investigate the functional role of HIF1A and its inhibitor PX-478 in modulating and potentially mitigating CAVD.

## Data availability statement

The raw data supporting the conclusions of this article will be made available by the authors, without undue reservation.

## Author contributions

MTS: executed the study, conducted all experiments, experimental and data analysis, and prepared the manuscript. NV-R: helped in experimental analysis and manuscript preparation. BV: helped in experimental analysis. HJ: co-principal investigator and provided supervision in all aspects of the study. AY: principal investigator and provided supervision in all aspects of the study. All authors contributed to the article and approved the submitted version.
